# Combining stereotactic radiosurgery and systemic therapy for brain metastases: a potential role for temozolomide

**DOI:** 10.3389/fonc.2012.00099

**Published:** 2012-08-09

**Authors:** Matthew E. Hardee, Silvia C. Formenti

**Affiliations:** ^1^Department of Radiation Oncology, New York University Langone Medical CenterNew York, NY, USA; ^2^Department of Medical Oncology, New York University Langone Medical CenterNew York, NY, USA

**Keywords:** brain metastases, temozolomide, radiosurgery

## Abstract

Brain metastases are unfortunately very common in the natural history of many solid tumors and remain a life-threatening condition, associated with a dismal prognosis, despite many clinical trials aimed at improving outcomes. Radiation therapy options for brain metastases include whole brain radiotherapy (WBRT) and stereotactic radiosurgery (SRS). SRS avoids the potential toxicities of WBRT and is associated with excellent local control (LC) rates. However, distant intracranial failure following SRS remains a problem, suggesting that untreated intracranial micrometastatic disease is responsible for failure of treatment. The oral alkylating agent temozolomide (TMZ), which has demonstrated efficacy in primary malignant central nervous system tumors such as glioblastoma, has been used in early phase trials in the treatment of established brain metastases. Although results of these studies in established, macroscopic metastatic disease have been modest at best, there is clinical and preclinical data to suggest that TMZ is more efficacious at treating and controlling clinically undetectable intracranial micrometastatic disease. We review the available data for the primary management of brain metastases with SRS, as well as the use of TMZ in treating established brain metastases and undetectable micrometastatic disease, and suggest the role for a clinical trial with the aims of treating macroscopically visible brain metastases with SRS combined with TMZ to address microscopic, undetectable disease.

## Introduction

Among the 1,596,670 Americans diagnosed with cancer in 2011, between 9.6–50.0% depending will develop one or more brain metastases during the course of their disease (American Cancer Society, 2011). Radiation Therapy Oncology Group (RTOG) has performed multiple studies for patients with brain metastases, unfortunately with limited progress: brain metastasis remains a life-threatening condition, associated with a dismal prognosis.

Recursive partitioning analysis of the results from three RTOG trials (1979 through 1993) divided patients into three prognostic groups, with distinct outcomes: (1) patients with a Karnofsky Performance Status (KPS) >70 and age less than 65, a controlled primary and the brain as the only site of metastases had a median survival of 7.1 months; (2) patients with a KPS <70 had a median survival of 2.3 months; (3) all of the other patients had a median survival of 4.2 months (Gaspar et al., 1997). A more recent review of the RTOG database has resulted in a new prognostic classification called Graded Prognostic Assessment (GPA), which incorporates the number of intracranial metastases to already identified prognostic factors (Sperduto et al., 2008). Median survivals range from 2.6 months in the poorest prognostic group (age >60, KPS <70, >3 intracranial metastases, and extracranial metastases present) to 11 months in the most favorable prognostic group (age <50, KPS 90–100, 1 intracranial metastasis present, and absence of extracranial metastases). As the median survival for the most favorable group in either prognostic classification system continues to be less than 12 months, it is clear that research to improve this condition is much needed.

This review focuses on the outcomes of brain metastases treated with stereotactic radiosurgery (SRS), the use of temozolomide (TMZ) in the treatment of brain metastases, and proposes the addition of systemic treatment with TMZ, with the goal of altering the course of intracranial disease by treating micrometastases throughout the brain.

## The role of SRS in treatment of limited brain metastases

Traditionally, patients with brain metastases have been treated with whole brain radiation therapy (WBRT) over the course of 2–3 weeks. As the name implies, this treatment is directed at both macroscopically visible tumors as well as microscopic tumor cells throughout the brain, taking advantage of an increased susceptibility of normal brain cells to repair ionizing radiation-induced DNA damage compared to tumor cells (Goodhead, 1994), increasing median survivals from 1 to 2 months with corticosteroids alone to approximately 3–6 months (Patchell, 2003).

Acute side effects from WBRT are common and include alopecia, fatigue, skin erythema, otitis externa, nausea, and headache. Early delayed and late side effects include tanning of the scalp, alopecia, hearing loss, hypopituitarism, neurocognitive decline, behavioral changes, somnolence syndrome, ataxia, and leukoencephalopathy (Suh, 2010).

SRS is a technique of radiation therapy that allows delivery of a highly conformal dose of radiation to a precisely defined target in a single treatment session, using multiple beams that converge on the center of that target, while limiting dose to surrounding normal brain tissue because of the steep radiation dose gradient achieved. Several types of delivery methods are used in SRS, including cobalt-60–based machines (Leksell Gamma Knife), linear accelerators, and cyclotrons. Potential benefits for SRS in the treatment of patients with limited brain metastases include not interrupting systemic chemotherapy, avoidance of alopecia, less fatigue, avoiding cognitive decline, convenience of a single treatment session, and avoidance of leukoencephalopathy (Elliott et al., 2011).

For patients with brain metastases, SRS offers a minimally invasive treatment that provides excellent local control (LC) rates. Numerous studies have shown the benefit of SRS on overall survival (OS) and LC. In RTOG protocol 9508, Andrews et al. treated 333 patients with 1–3 brain metastases, randomizing between WBRT alone and WBRT + SRS (Andrews et al., 9508). The addition of SRS improved LC rates (43% higher risk of local recurrence without SRS) and median OS in patients with a single brain metastasis (6.5 vs 4.9 months). Improved performance status and decreased steroid use were also reported at six months. Kondziolka et al. also reported a study of 27 patients randomized to WBRT with or without SRS for patients for two to four brain metastases. The trial was closed early after a significant interim benefit in LC for WBRT + SRS arm was seen (0 vs 92%) (Kondziolka et al., 1999).

The use of SRS alone for patients with limited brain metastases has been increasing, avoiding use of upfront WBRT. The need for WBRT in addition to SRS has been examined in several prospective randomized trials. Aoyama et al. reported a prospective trial comparing SRS with and without WBRT in 132 patients with one to four brain metastases, with no improvement seen in overall or high-functioning survival with the addition of WBRT (Aoyama et al., 2006). While distant intracranial failure and the need for salvage treatment were higher with SRS alone, rates of neurologic death were similar between the two groups (22.8 vs 19.3%). Chang et al. randomized 58 patients to SRS alone vs SRS plus WBRT in patients with one to three brain metastases (Chang et al., 2009). The trial was stopped early due to a greater decline in learning and memory in the group that received WBRT (52 vs 25%). While more patients in the SRS alone group required salvage therapies, they also experienced longer median OS and improved long-term cognitive functioning.

Table 1 summarizes published series using SRS alone for the treatment of limited brain metastases. While there are no randomized trials vs WBRT, SRS provides excellent local rates, and comparable OS. Collectively, rates of OS range from 7–13.8 months and LC from 76–94%. Current recommendations for SRS include treatment of one to four brain metastases, which are less than 3–4 cm in size. Ideally, patients have controlled extracranial metastases, excellent performance status, and little to no mass effect or clinically significant edema associated with their metastases. A recent ASTRO recommendation regarding the use of radiotherapeutic management for newly diagnosed brain metastases has recently been published (Tsao et al., 2012).

**Table 1:** **Outcomes following SRS alone for limited brain metastases**.

**Study References**	**Study**	**Patients (n)**	**Lesions (n)**	**Crude LC (%)**	**1yr LC (%)**	**2yr LC (%)**	**Median OS (mos)**	**Neurologic death (%)**
Aoyama et al., 2006	PRCT	67	1–4	NR	72.5	NR	7.5	19.3
Lutterbach et al., 2003	PO	101	1–3	92	91	79	7.6	NR
Williams et al., 2009	PO	273	1–2	76	NR	NR	10.3	NR
Chitapanarux et al., 2003	PO	41	1–4	NR	68	NR	10	12
Gerosa et al., 2002	R	804	1–3	93	NR	NR	13.5	15.6
Kihlstrom et al., 1993	R	160	1–5	94	NR	NR	7	9.7
Hasegawa et al., 2003	R	172	1–4	87	79	75	8	16.5
Flickinger et al., 1994	R	116	1	85	NR	67	11	NR
Petrovich et al., 2002	R	458	1–5	NR	90	NR	9	32.6
Elliott et al., 2011	R	109	1–3	93	93	89	13.8	11.9

PRCT, prospective, randomized controlled trial; PO, prospective observational trial; R, retrospective series; NR, not reported.

Despite adequate LC, the dominant pattern of failure in many of these series remains distant intracranial progression. Chitapanarux et al. reported a “regional” progression rate (outside treated lesions) of 49% at a median follow-up of 7 months. In a prospective series of 101 patients, Lutterbach et al found distant brain progression in 34%, 47%, and 78% at 6, 12, and 24 months, respectively, in patients with brain metastases treated with SRS only. In their randomized trial of SRS vs SRS + WBRT, Aoyama et al. reported a 12 month actuarial rate of developing new intracranial metastases of 64% in patients treated with SRS alone. Clearly, an opportunity exists to improve these outcomes, decreasing the need for salvage treatment in patients failing regionally.

## SRS treatment alone for more extensive intracranial metastases

While SRS has traditionally been recommended for patients with four or less intracranial lesions, several groups have reported on the use of SRS alone in patients with more extensive intracranial disease. Bhatnagar et al. reported on the University of Pittsburgh experience treating four or more intracranial metastases (Bhatnagar et al., 2006). In 205 patients with a median of five intracranial metastases, they demonstrated that, not only was SRS feasible in this patient population, but also that SRS seemed to provide a survival advantage when compared to historic controls in this patient population. Further, total treatment volume (and not number of metastases) was the only significant factor associated with local recurrence on multivariate analysis.

In a small retrospective study, Lee et al. reported on 15 patients with one to twelve non-germ cell epithelial ovarian cancer brain metastases treated with either SRS alone or WBRT alone (Lee et al., 2008). While some details of treatment and intergroup differences were lacking, the SRS arm had a significantly improved median survival compared to the WBRT arm (29 vs 6 months). Suzuki et al. reported on 24 patients with 10 or more intracranial metastases treated SRS alone (Suzuki et al., 2000). While median survival was only 2.8 months, none of the patients died of neurologic deterioration. In a larger series of 323 patients with multiple brain metastases treated with SRS alone, Chang et al. reported outcomes based on the number of brain lesions (Chang et al., 2010). Median survival and local tumor control rates were not significantly different between patients with 1–5, 6–10, 11–15, or greater than 15 lesions, although patients initially treated for >15 lesions had higher risk of distant intracranial failure.

The saftey of SRS treatment of extensive intracranial metastatic disease has also been examined. Yamamoto et al. reported on 92 SRS procedures for 80 patients with 10 more treated lesions per treatment session (Yamamoto et al., 2002). The cumulative whole brain dose was found to be equivalent to approximately 12 Gy WBRT, well below WBRT doses generally prescribed for brain metastases (30–40 Gy) and below doses associated with unacceptable toxicity.

## Mechanism of action of TMZ and experience in gliomas

TMZ is an orally-available alkylating agent already FDA-approved for treatment of malignant gliomas. Alkylating agents chemically modify a cell's DNA by addition of an alkyl group (a chemical functional group), irreparably damaging the tumor DNA with resultant killing of cancer cells. Once absorbed, TMZ requires conversion to its active metabolite MTIC, which occurs spontaneously under physiologic conditions (Danson and Middleton, 2001). Because of its small molecular weight (194 Da), TMZ easily penetrates the blood-brain-barrier (BBB). It is administered orally, with almost complete absorption when taken on an empty stomach. The most commonly seen side effects of TMZ include hematologic toxicities (thrombocytopenia and granulocytopenia particularly), fatigue, nausea, vomiting, and constipation (Lens and Eisen, 2003).

TMZ has been studied for the treatment of anaplastic gliomas (WHO grade III) and glioblastoma (GBM; WHO grade IV). In a seminal trial reported by Stupp et al. the addition of TMZ to radiation therapy in the postoperative treatment of GBM increased median OS from 12.1 to 14.6 months (Stupp et al., 2009). An improvement was also seen in 2 year survival, with 26.5% of TMZ plus radiotherapy patients surviving, compared to 10.4% of patients treated with radiotherapy alone.

## Prediction of response to TMZ by MGMT status

Interestingly, in GBM the effectiveness of this drug is associated with silencing of a specific DNA-repair enzyme, O^6^-methylguanine–DNA methyltransferase (MGMT). MGMT rapidly reverses DNA alkylation at the O^6^ position of guanine by transferring the alkyl group to the active site of the enzyme itself (Pegg, 2000). Lack of MGMT in the cell allows accumulation of O^6^-alkylguanine in the DNA, which triggers mismatch repair, thereby inducing DNA damage signaling and eventual cell death (Weller et al., 2010). A major mechanism of MGMT regulation is via promoter methylation. The MGMT promoter lacks constitutive regulatory elements such as TATA box and CAT box. It does, however, contain a CpG island containing a high density of CG dinucleotides, which are unmethylated in normal tissues. In an aberrantly methylated state, methyl-CpG-binding proteins (such as MeCP2 and MBD2), can bind to the promoter, leading to alterations of chromatin structure and preventing binding of transcription factors and silencing of the gene, leading to resistance to alkylating agents (Nakagawachi et al., 2003).

The MGMT promoter methylation status is the strongest prognostic factor for outcome in patients with newly diagnosed glioblastoma and is a powerful predictor of response to alkylating chemotherapy. Hegi et al. further analyzed the MGMT methylation status of tumors in the EORTC/NCIC trial reported by Stupp (Hegi et al., 2005). The 2 year and 5 year survival rates in patients with a methylated MGMT promoter treated with concomitant and adjuvant TMZ were 49 and 14%, respectively, while the corresponding figures for patients initially treated with radiotherapy only were 24 and 5%. Of patients with an unmethylated MGMT promoter, 15 and 8% were alive at 2 years and 5 years, respectively, after treatment with combined chemoradiotherapy, compared with 2 and 0% in those initially treated with radiotherapy alone (Table 2).

**Table 2:** **Results of TMZ treatment for established brain metastases**.

**References**	**Phase**	**Histology**	**N**	**Dose/Schedule**	**Responses**
Schadendorf et al., 2006	II	Melanoma	45	125–150 mg/m^2^	2 PR (4%)
				D1–7, 15–21 q28d	5 SD (11%)
Siena et al., 2010	II	Melanoma, breast, NSCLC	157	150 mg/m^2^	1 CR
				D1–7, 15–21, q28 or q35d	9 PR (6%)
					31 SD (20%)
Christodoulou et al., 2001	II	Solid tumors	27	150 mg/m^2^	1 PR (4%)
				5 days q28d	4 SD (17%)
					Neuro status improved in 37% patients
Omuro et al., 2006	I	Recurrent/ progressive brain mets	21	150 mg/m^2^	2 PR (11%)
				D1–7, 15–21 q28d	6 SD (33%)
				Vinorelbine D1 and D8	
Iwamoto et al., 2008	II	Solid tumors	38	150 mg/m^2^	1 CR (3%)
				D1–7, 15–21 q28d	1 PR (3%)
				Vinorelbine D1 and D8	5 SD (13%)
Rivera et al., 2006	I	Breast	24	75 mg/m^2^	1 CR (4%)
				Capecitabine 1800 mg/m^2^	3 PR (13%)
				D1–5, 8–12 q21d	
Christodoulou et al., 2005	II	Solid tumors	32	150–200 mg/m^2^	1 CR (3%)
				D1–5	9 PR (28%)
				CDDP 75 mg/m^2^	5 SD (16%)
				D1 q28d	
Abrey et al., 2001	II	Recurrent brain mets	34	150–200 mg/m^2^	2 PR (6%)
				D1–5 q28d	5 SD (15%)

PR, partial response; CR, complete response; SD, stable disease.

## Disappointing results with the use of TMZ in patients with established brain metastases

The use of traditional chemotherapeutic agents in the treatment of brain metastases has been met with much skepticism, largely due to concerns over the large size or hydrophilic nature of these compounds and their inability to cross the BBB. As noted above, TMZ has little difficulty crossing the BBB and has demonstrated efficacy against primary CNS tumors. For these reasons, the TMZ alone or in combination with other chemotherapeutic agents has been used to treat brain metastases in several early phase clinical trials with modest results (Table 2). Overall response rates are not overwhelming and range from 4 to 13%, although freedom from progression rates range from 15 to 44%.

TMZ has also been combined with WBRT for the treatment of established brain metastases, including two randomized trials, with equally disappointing results (Table 3). It should be noted, however, that the majority of these patients were heavily pre-treated and harbor clinically apparent, often symptomatic, brain metastases. In the 2 randomized trials, there appeared to be some benefit to adding TMZ to WBRT. Antonadou et al. reported an increased objective response rate of 96% when TMZ was added to WBRT, compared to 67% with WBRT alone. This was also associated with a decrease in the need for steroid use 2 months following WBRT (67 vs 91%). While Verger et al. did not find a difference in PFS or OS, the addition of TMZ to WBRT did decrease the incidence of neurologic death (69 vs 41%).

**Table 3:** **Results with TMZ and WBRT for established brain metastases**.

**References**	**Histology**	**Phase**	**n**	**Dose/Schedule**	**Response**
Mikkelsen et al., 2010	Solid tumors	I/II	17	95 mg/m^2^	3 PR (18%)
				WBRT 30 Gy	10 SD (59%)
					6 mo PFS 18%; median PFS 2.4 mo
Kouvaris et al., 2007	Solid tumors	II	33	60 mg/m^2^	7 CR (21%)
				D1–15	11 PR (33%)
				WBRT 36 Gy	5 SD (15%)
					Median OS 12
Addeo et al., 2008	Breast, NSCLC	II	27	75 mg/m^2^	2 CR (7%)
				D1–10 q21–28d	11 PR (41%)
				WBRT 30 Gy	Median PFS 6 mos, OS 8.8 mos
Verger et al., 2005	Solid tumors	II^*^	82	±75 mg/m^2^	Randomized ± TMZ
				WBRT 30 Gy	No difference in PFS or OS
					Neuro death decreased with TMZ (69% vs 41%)
Addeo et al., 2007	Solid tumors	II	59	75 mg/m^2^	5 CR (8%)
				D1–10	21 PR (36%)
				WBRT 30 Gy	18 SD (31%)
					Improved QoL with TMZ
Antonadou et al., 2002	Solid tumors	II^*^	43	±75 mg/m^2^	ORR increased (96% vs 67%)
				D1–5	Decreased need for steroids 2 mos after treatment (67% vs 91%)
				WBRT 40 Gy	
Margolin et al., 2002	Melanoma	II	31	75 mg/m^2^	1 CR (3%)
				Daily × 6 weeks	2 PR (6%)
				WBRT 30 Gy	

* Randomized phase II trial; PR, partial response; CR, complete response; SD, stable disease; ORR, objective response rate; PFS, progression free survival; OS, overall survival.

## Increased effect of chemotherapy in microscopic versus detectable brain metastases

Recent preclinical evidence suggests a role for a different strategy: after radiosurgery of lesions visible on neuroimaging, treatment of non-radiologically visible micro-metastases might ultimately be more successful and delay recurrence at distant sites in the brain. Dr. Patricia Steeg at NCI has developed data supportive of this hypothesis and has identified a series of drugs that have shown some promise in this direction (Gril et al., 2008, 2010, 2011; Qian et al., 2011; Steeg et al., 2011). For example, in the 231-BR brain metastasis model of the human breast cancer cell line MDA-MB-231, Palmieri et al. demonstrated that the histone deacetylase (HDAC) inhibitor Vorinostat was effective in preventing new metastases from forming, but had significantly decreased efficacy against established macrometastases (Palmieri et al., 2009).

Further, clinical evidence exists supporting this hypothesis that, while TMZ fails once metastases have established, it can be an effective adjuvant treatment of microscopic intracranial metastatic disease (Robins et al., 2006). Choong et al. reported on the use of TMZ and irinotecan as second-line systemic therapy for non-small cell lung cancer (Choong et al., 2006). The authors reported a CNS-relapse rate of only 8%, which is particularly favorable compared to brain metastases rates of >30% in comparable patient populations (Tannehill et al., 1997). Additionally, an earlier study reported a CNS metastasis rate of only 3% with the use of TMZ alone as salvage therapy for pre-treated non-small cell lung cancer patients (Adonizio et al., 2002). Thus, it seems TMZ may be effective in addressing intracranial micrometastatic disease and delaying clinical manifestation of these metastases.

## Opportunity for clinical trial combining SRS and TMZ

We believe the above data reveals an ideal opportunity for combining SRS treatment of clinically apparent brain metastases and systemic treatment of micrometastatic disease with TMZ. Available data suggests that treatment of up to 10 brain metastases is feasible and safe, and we will pursue such an approach to avoid (or delay) the use of WBRT. While macrometastases will be treated with SRS, systemic treatment with TMZ should address micrometastases and delay or prevent establishment of further clinically detectable macrometastases.

Further, as noted above, MGMT status is a powerful predictor of outcomes in response to treatment in GBM. Recently, a role for MGMT status in determining treatment outcomes in brain metastases has also been described. Wu et al. reported on 86 brain metastases from non-small cell lung cancer (Wu et al., 2010). Both univariate and multivariate analyses showed that median OS was significantly longer in patients with positive MGMT expression in brain metastases (16.5 versus 3.5 months, *P* < 0.001).

It is possible that only low-MGMT expressing metastases respond to the drug. To explore this hypothesis, efforts to collect the original tumor will be undertaken, to measure MGMT expression in the primary and investigate its role in predicting response to TMZ after SRS for brain metastasis.

## Conclusions

LC of established brain metastases treated with SRS has been excellent; however eventual failure “distantly” in brain is unfortunately extremely common. While results of TMZ for established intracranial metastases is not overwhelming, there is clinical and preclinical evidence to suggest it is more effective at addressing micrometastatic disease and preventing clinical manifestation of macrometastases. The proposed trial combining SRS with TMZ represents a model for testing the modification of the course of clinical disease in the brain. While this approach is not new, it has never been tested in a prospective, rational, and targeted fashion.

### Conflict of interest statement

The authors declare that the research was conducted in the absence of any commercial or financial relationships that could be construed as a potential conflict of interest.

## References

[B1] American Cancer Society (2011). Cancer Facts, and Figures 2011. Available online at: http://www.cancer.org/acs/groups/content/@epidemiologysurveilance/documents/document/acspc-029771.pdf 20848386

[B2] Abrey L. E. Olson J. D. Raizer J. J. Mack M. Rodavitch A. Boutros D. Y. Malkin M. G. (2001). A phase II trial of temozolomide for patients with recurrent or progressive brain metastases. J. Neurooncol. 53, 259–265 10.1023/A:1012226718323 11718258

[B3] Addeo R. Caraglia M. Faiola V. Capasso E. Vincenzi B. Montella L. Guarrasi R. Caserta L. Del Prete S. (2007). Concomitant treatment of brain metastasis with whole brain radiotherapy [WBRT] and temozolomide [TMZ] is active and improves quality of life. BMC Cancer 7, 18 10.1186/1471-2407-7-18 17254350 PMC1794253

[B4] Addeo R. De Rosa C. Faiola V. Leo L. Cennamo G. Montella L. Guarrasi R. Vincenzi B. Caraglia M. Del Prete S. (2008). Phase 2 trial of temozolomide using protracted low-dose and whole-brain radiotherapy for nonsmall cell lung cancer and breast cancer patients with brain metastases. Cancer 113, 2524–2531 10.1002/cncr.23859 18798231

[B5] Adonizio C. S. Babb J. S. Maiale C. Huang C. Donahue J. Millenson M. M. Hosford M. Somer R. Treat J. Sherman E. Langer C. J. (2002). Temozolomide in non-small-cell lung cancer: preliminary results of a phase II trial in previously treated patients. Clin. Lung Cancer 3, 254–258 14662033 10.3816/clc.2002.n.009

[B6] Andrews D. W. Scott C. B. Sperduto P. W. Flanders A. E. Gaspar L. E. Schell M. C. Werner-Wasik M. Demas W. Ryu J. Bahary J. P. Souhami L. Rotman M. Mehta M. P. Curran W. J. Jr. (2004). Whole brain radiation therapy with or without stereotactic radiosurgery boost for patients with one to three brain metastases: phase III results of the RTOG (9508). randomised trial. Lancet 363, 1665–1672 10.1016/S0140-6736(04)16250-8 15158627

[B7] Antonadou D. Paraskevaidis M. Sarris G. Coliarakis N. Economou I. Karageorgis P. Throuvalas N. (2002). Phase II randomized trial of temozolomide and concurrent radiotherapy in patients with brain metastases. J. Clin. Oncol. 20, 3644–3650 10.1200/JCO.2002.04.140 12202665

[B8] Aoyama H. Shirato H. Tago M. Nakagawa K. Toyoda T. Hatano K. Kenjyo M. Oya N. Hirota S. Shioura H. Kunieda E. Inomata T. Hayakawa K. Katoh N. Kobashi G. (2006). Stereotactic radiosurgery plus whole-brain radiation therapy vs stereotactic radiosurgery alone for treatment of brain metastases: a randomized controlled trial. JAMA 295, 2483–2491 10.1001/jama.295.21.2483 16757720

[B9] Bhatnagar A. K. Flickinger J. C. Kondziolka D. Lunsford L. D. (2006). Stereotactic radiosurgery for four or more intracranial metastases. Int. J. Radiat. Oncol. Biol. Phys. 64, 898–903 10.1016/j.ijrobp.2005.08.035 16338097

[B10] Chang E. L. Wefel J. S. Hess K. R. Allen P. K. Lang F. F. Kornguth D. G. Arbuckle R. B. Swint J. M. Shiu A. S. Maor M. H. Meyers C. A. (2009). Neurocognition in patients with brain metastases treated with radiosurgery or radiosurgery plus whole-brain irradiation: a randomised controlled trial. Lancet Oncol. 10, 1037–1044 10.1016/S1470-2045(09)70263-3 19801201

[B11] Chang W. S. Kim H. Y. Chang J. W. Park Y. G. Chang J. H. (2010). Analysis of radiosurgical results in patients with brain metastases according to the number of brain lesions: is stereotactic radiosurgery effective for multiple brain metastases? J. Neurosurg. 113(Suppl.), 73–78 21121789 10.3171/2010.8.GKS10994

[B12] Chitapanarux I. Goss B. Vongtama R. Frighetto L. De Salles A. Selch M. Duick M. Solberg T. Wallace R. Cabatan-Awang C. Ford J. (2003). Prospective study of stereotactic radiosurgery without whole brain radiotherapy in patients with four or less brain metastases: incidence of intracranial progression and salvage radiotherapy. J. Neurooncol. 61, 143–149 10.1023/A:1022173922312 12622453

[B13] Choong N. W. Mauer A. M. Hoffman P. C. Rudin C. M. Winegarden J. D. 3rd Villano J. L. Kozloff M. Wade J. L. 3rd Sciortino D. F. Szeto L. Vokes E. E. (2006). Phase II trial of temozolomide and irinotecan as second-line treatment for advanced non-small cell lung cancer. J. Thorac. Oncol. 1, 245–251 17409864 10.1016/s1556-0864(15)31575-6

[B14] Christodoulou C. Bafaloukos D. Kosmidis P. Samantas E. Bamias A. Papakostas P. Karabelis A. Bacoyiannis C. Skarlos D. V. (2001). Phase II study of temozolomide in heavily pretreated cancer patients with brain metastases. Ann. Oncol. 12, 249–254 11300333 10.1023/a:1008354323167

[B15] Christodoulou C. Bafaloukos D. Linardou H. Aravantinos G. Bamias A. Carina M. Klouvas G. Skarlos D. (2005). Temozolomide (TMZ) combined with cisplatin (CDDP) in patients with brain metastases from solid tumors: a Hellenic Cooperative Oncology Group (HeCOG) Phase II study. J. Neurooncol. 71, 61–65 10.1007/s11060-004-9176-0 15719277

[B16] Danson S. J. Middleton M. R. (2001). Temozolomide: a novel oral alkylating agent. Expert Rev. Anticancer Ther. 1, 13–19 12113120 10.1586/14737140.1.1.13

[B17] Elliott R. E. Rush S. C. Morsi A. Mehta N. Spriet J. Narayana A. Donahue B. Parker E. C. Golfinos J. G. (2011). Local control of newly diagnosed and distally recurrent, low-volume brain metastases with fixed-dose (20 gy) gamma knife radiosurgery. Neurosurgery 68, 921–931 discussion: 931. 10.1227/NEU.0b013e318208f58e 21221034

[B18] Flickinger J. C. Kondziolka D. Lunsford L. D. Coffey R. J. Goodman M. L. Shaw E. G. Hudgins W. R. Weiner R. Harsh G. R. Sneed P. K. Larson D. A. (1994). A multi-institutional experience with stereotactic radiosurgery for solitary brain metastasis. Int. J. Radiat. Oncol. Biol. Phys. 28, 797–802 8138431 10.1016/0360-3016(94)90098-1

[B19] Gaspar L. Scott C. Rotman M. Asbell S. Phillips T. Wasserman T. McKenna W. G. Byhardt R. (1997). Recursive partitioning analysis (RPA) of prognostic factors in three Radiation Therapy Oncology Group (RTOG) brain metastases trials. Int. J. Radiat. Oncol. Biol. Phys. 37, 745–751 9128946 10.1016/s0360-3016(96)00619-0

[B20] Gerosa M. Nicolato A. Foroni R. Zanotti B. Tomazzoli L. Miscusi M. Alessandrini F. Bricolo A. (2002). Gamma knife radiosurgery for brain metastases: a primary therapeutic option. J. Neurosurg. 97, 515–524 10.1038/jcbfm.1994.25 12507088

[B21] Goodhead D. T. (1994). Initial events in the cellular effects of ionizing radiations: clustered damage in DNA. Int. J. Radiat. Biol. 65, 7–17 7905912 10.1080/09553009414550021

[B22] Gril B. Palmieri D. Bronder J. L. Herring J. M. Vega-Valle E. Feigenbaum L. Liewehr D. J. Steinberg S. M. Merino M. J. Rubin S. D. Steeg P. S. (2008). Effect of lapatinib on the outgrowth of metastatic breast cancer cells to the brain. J. Natl. Cancer Inst. 100, 1092–1103 10.1093/jnci/djn216 18664652 PMC2575427

[B23] Gril B. Evans L. Palmieri D. Steeg P. S. (2010). Translational research in brain metastasis is identifying molecular pathways that may lead to the development of new therapeutic strategies. Eur. J. Cancer 46, 1204–1210 10.1016/j.ejca.2010.02.033 20303257 PMC2858326

[B24] Gril B. Palmieri D. Qian Y. Smart D. Ileva L. Liewehr D. J. Steinberg S. M. Steeg P. S. (2011). Pazopanib reveals a role for tumor cell B-Raf in the prevention of HER2+ breast cancer brain metastasis. Clin. Cancer Res. 17, 142–153 10.1158/1078-0432.CCR-10-1603 21081656 PMC3059742

[B25] Hasegawa T. Kondziolka D. Flickinger J. C. Germanwala A. Lunsford L. D. (2003). Brain metastases treated with radiosurgery alone: an alternative to whole brain radiotherapy? Neurosurgery 52, 1318–1326 discussion: 1326. 12762877 10.1227/01.neu.0000064569.18914.de

[B26] Hegi M. E. Diserens A. C. Gorlia T. Hamou M. F. de Tribolet N. Weller M. Kros J. M. Hainfellner J. A. Mason W. Mariani L. Bromberg J. E. Hau P. Mirimanoff R. O. Cairncross J. G. Janzer R. C. Stupp R. (2005). MGMT gene silencing and benefit from temozolomide in glioblastoma. N. Engl. J. Med. 352, 997–1003 10.1056/NEJMoa043331 15758010

[B27] Iwamoto F. M. Omuro A. M. Raizer J. J. Nolan C. P. Hormigo A. Lassman A. B. Gavrilovic I. T. Abrey L. E. (2008). A phase II trial of vinorelbine and intensive temozolomide for patients with recurrent or progressive brain metastases. J. Neurooncol. 87, 85–90 10.1007/s11060-007-9491-3 17987262

[B28] Kihlstrom L. Karlsson B. Lindquist C. (1993). Gamma Knife surgery for cerebral metastases. Implications for survival based on 16 years experience. Stereotact. Funct. Neurosurg. 61(Suppl. 1), 45–50 8115755 10.1159/000100659

[B29] Kondziolka D. Patel A. Lunsford L. D. Kassam A. Flickinger J. C. (1999). Stereotactic radiosurgery plus whole brain radiotherapy versus radiotherapy alone for patients with multiple brain metastases. Int. J. Radiat. Oncol. Biol. Phys. 45, 427–434 10487566 10.1016/s0360-3016(99)00198-4

[B30] Kouvaris J. R. Miliadou A. Kouloulias V. E. Kolokouris D. Balafouta M. J. Papacharalampous X. N. Vlahos L. J. (2007). Phase II study of temozolomide and concomitant whole-brain radiotherapy in patients with brain metastases from solid tumors. Onkologie 30, 361–366 10.1159/0000102557 17596744

[B31] Lee Y. K. Park N. H. Kim J. W. Song Y. S. Kang S. B. Lee H. P. (2008). Gamma-knife radiosurgery as an optimal treatment modality for brain metastases from epithelial ovarian cancer. Gynecol. Oncol. 108, 505–509 10.1016/j.ygyno.2007.11.027 18191184

[B32] Lens M. B. Eisen T. G. (2003). Systemic chemotherapy in the treatment of malignant melanoma. Expert Opin. Pharmacother. 4, 2205–2211 10.1517/14656566.4.12.2205 14640919

[B33] Lutterbach J. Cyron D. Henne K. Ostertag C. B. (2003). Radiosurgery followed by planned observation in patients with one to three brain metastases. Neurosurgery 52, 1066–1073 discussion: 1073–1074. 12699548

[B34] Margolin K. Atkins B. Thompson A. Ernstoff S. Weber J. Flaherty L. Clark I. Weiss G. Sosman J. Smith W. II Dutcher P. Gollob J. Longmate J. Johnson D. (2002). Temozolomide and whole brain irradiation in melanoma metastatic to the brain: a phase II trial of the Cytokine Working Group. J. Cancer Res. Clin. Oncol. 128, 214–218 10.1007/s00432-002-0323-8 11935312 PMC12164456

[B35] Mikkelsen T. Anderson J. Doyle T. J. Croteau D. Avedissian R. Ryu S. Schultz L. (2010). Phase I/II dose escalation trial of concurrent temozolomide and whole brain radiation therapy for multiple brain metastasis. J. Neurooncol. 100, 241–247 10.1007/s11060-010-0187-8 20431907

[B36] Nakagawachi T. Soejima H. Urano T. Zhao W. Higashimoto K. Satoh Y. Matsukura S. Kudo S. Kitajima Y. Harada H. Furukawa K. Matsuzaki H. Emi M. Nakabeppu Y. Miyazaki K. Sekiguchi M. Mukai T. (2003). Silencing effect of CpG island hypermethylation and histone modifications on O6-methylguanine-DNA methyltransferase (MGMT) gene expression in human cancer. Oncogene 22, 8835–8844 10.1038/sj.onc.1207183 14647440

[B37] Omuro A. M. Raizer J. J. Demopoulos A. Malkin M. G. Abrey L. E. (2006). Vinorelbine combined with a protracted course of temozolomide for recurrent brain metastases: a phase I trial. J. Neurooncol. 78, 277–280 10.1007/s11060-005-9095-8 16614943

[B38] Palmieri D. Lockman P. R. Thomas F. C. Hua E. Herring J. Hargrave E. Johnson M. Flores N. Qian Y. Vega-Valle E. Taskar K. S. Rudraraju V. Mittapalli R. K. Gaasch J. A. Bohn K. A. Thorsheim H. R. Liewehr D. J. Davis S. Reilly J. F. Walker R. Bronder J. L. Feigenbaum L. Steinberg S. M. Camphausen K. Meltzer P. S. Richon V. M. Smith Q. R. Steeg P. S. (2009). Vorinostat inhibits brain metastatic colonization in a model of triple-negative breast cancer and induces DNA double-strand breaks. Clin. Cancer Res. 15, 6148–6157 10.1158/1078-0432.CCR-09-1039 19789319 PMC7356672

[B39] Patchell R. A. (2003). The management of brain metastases. Cancer Treat. Rev. 29, 533–540 10.1016/S0305-7372(03)00105-1 14585263

[B40] Pegg A. E. (2000). Repair of O(6)-alkylguanine by alkyltransferases. Mutat. Res. 462, 83–100 10.1016/S1383-5742(00)00017-X 10767620

[B41] Petrovich Z. Yu C. Giannotta S. L. O'Day S. Apuzzo M. L. (2002). Survival and pattern of failure in brain metastasis treated with stereotactic gamma knife radiosurgery. J. Neurosurg. 97, 499–506 10.3171/jns.2002.97.supplement5.0499 12507085

[B42] Qian Y. Hua E. Bisht K. Woditschka S. Skordos K. W. Liewehr D. J. Steinberg S. M. Brogi E. Akram M. M. Killian J. K. Edelman D. C. Pineda M. Scurci S. Degenhardt Y. Y. Laquerre S. Lampkin T. A. Meltzer P. S. Camphausen K. Steeg P. S. Palmieri D. (2011). Inhibition of Polo-like kinase 1 prevents the growth of metastatic breast cancer cells in the brain. Clin. Exp. Metastasis 28, 899–908 10.1007/s10585-011-9421-9 21953073 PMC7416514

[B43] Rivera E. Meyers C. Groves M. Valero V. Francis D. Arun B. Broglio K. Yin G. Hortobagyi G. N. Buchholz T. (2006). Phase I study of capecitabine in combination with temozolomide in the treatment of patients with brain metastases from breast carcinoma. Cancer 107, 1348–1354 10.1002/cncr.22127 16909414

[B44] Robins H. I. Traynor A. M. Mehta M. (2006). Temozolomide as prophylaxis for brain metastasis in non-small cell lung cancer. J. Thorac. Oncol. 1, 732–733 Author reply: 733. 17409946

[B45] Schadendorf D. Hauschild A. Ugurel S. Thoelke A. Egberts F. Kreissig M. Linse R. Trefzer U. Vogt T. Tilgen W. Mohr P. Garbe C. (2006). Dose-intensified bi-weekly temozolomide in patients with asymptomatic brain metastases from malignant melanoma: a phase II DeCOG/ADO study. Ann. Oncol. 17, 1592–1597 10.1093/annonc/mdl148 17005632

[B46] Siena S. Crino L. Danova M. Del Prete S. Cascinu S. Salvagni S. Schiavetto I. Vitali M. Bajetta E. (2010). Dose-dense temozolomide regimen for the treatment of brain metastases from melanoma, breast cancer, or lung cancer not amenable to surgery or radiosurgery: a multicenter phase II study. Ann. Oncol. 21, 655–661 10.1093/annonc/mdp343 19767314 PMC2826096

[B47] Sperduto P. W. Berkey B. Gaspar L. E. Mehta M. Curran W. (2008). A new prognostic index and comparison to three other indices for patients with brain metastases: an analysis of 1,960 patients in the RTOG database. Int. J. Radiat. Oncol. Biol. Phys. 70, 510–514 10.1016/j.ijrobp.2007.06.074 17931798

[B48] Steeg P. S. Camphausen K. A. Smith Q. R. (2011). Brain metastases as preventive and therapeutic targets. Nat. Rev. Cancer 11, 352–363 10.1038/nrc3053 21472002 PMC7351203

[B49] Stupp R. Hegi M. E. Mason W. P. van den Bent M. J. Taphoorn M. J. Janzer R. C. Ludwin S. K. Allgeier A. Fisher B. Belanger K. Hau P. Brandes A. A. Gijtenbeek J. Marosi C. Vecht C. J. Mokhtari K. Wesseling P. Villa S. Eisenhauer E. Gorlia T. Weller M. Lacombe D. Cairncross J. G. Mirimanoff R. O. (2009). Effects of radiotherapy with concomitant and adjuvant temozolomide versus radiotherapy alone on survival in glioblastoma in a randomised phase III study: 5-year analysis of the EORTC-NCIC trial. Lancet Oncol. 10, 459–466 10.1016/S1470-2045(09)70025-7 19269895

[B50] Suh J. H. (2010). Stereotactic radiosurgery for the management of brain metastases. N. Engl. J. Med. 362, 1119–1127 10.1056/NEJMct0806951 20335588

[B51] Suzuki S. Omagari J. Nishio S. Nishiye E. Fukui M. (2000). Gamma knife radiosurgery for simultaneous multiple metastatic brain tumors. J. Neurosurg. 93(Suppl. 3), 30–31 10.3171/jns.2000.93.supplement3.0030 11143258

[B52] Tannehill S. P. Mehta M. P. Larson M. Storer B. Pellet J. Kinsella T. J. Schiller J. H. (1997). Effect of amifostine on toxicities associated with sequential chemotherapy and radiation therapy for unresectable non-small-cell lung cancer: results of a phase II trial. J. Clin. Oncol. 15, 2850–2857 9256128 10.1200/JCO.1997.15.8.2850

[B53] Tsao M. N. Rades D. Wirth A. Lo S. S. Danielson B. L. Gaspar L. E. Sperduto P. W. Vogelbaum M. A. Radawski J. D. Wang J. Z. Gillin M. T. Mohideen N. Hahn C. A. Chang E. L. (2012). Radiotherapeutic and surgical management for newly diagnosed brain metastasis (es): An American Society for Radiation Oncology evidence-based guideline. Pract. Radiat. Oncol. 2, 210–225 10.1016/j.prro.2011.12.004 PMC3808749 25925626

[B54] Verger E. Gil M. Yaya R. Vinolas N. Villa S. Pujol T. Quinto L. Graus F. (2005). Temozolomide and concomitant whole brain radiotherapy in patients with brain metastases: a phase II randomized trial. Int. J. Radiat. Oncol. Biol. Phys. 61, 185–191 10.1016/j.ijrobp.2004.04.061 15629610

[B55] Weller M. Stupp R. Reifenberger G. Brandes A. A. van den Bent M. J. Wick W. Hegi M. E. (2010). MGMT promoter methylation in malignant gliomas: ready for personalized medicine? Nat. Rev. Neurol. 6, 39–51 10.1038/nrneurol.2009.197 19997073

[B56] Williams B. J. Suki D. Fox B. D. Pelloski C. E. Maldaun M. V. Sawaya R. E. Lang F. F. Rao G. (2009). Stereotactic radiosurgery for metastatic brain tumors: a comprehensive review of complications. J. Neurosurg. 111, 439–448 10.3171/2008.11.JNS08984 19301968

[B57] Wu P. F. Kuo K. T. Kuo L. T. Lin Y. T. Lee W. C. Lu Y. S. Yang C. H. Wu R. M. Tu Y. K. Tasi J. C. Tseng H. M. Tseng S. H. Cheng A. L. Lin C. H. (2010). O(6)-Methylguanine-DNA methyltransferase expression and prognostic value in brain metastases of lung cancers. Lung Cancer 68, 484–490 10.1016/j.lungcan.2009.08.010 19740564

[B58] Yamamoto M. Ide M. Nishio S. Urakawa Y. (2002). Gamma Knife radiosurgery for numerous brain metastases: is this a safe treatment? Int. J. Radiat. Oncol. Biol. Phys. 53, 1279–1283 12128130 10.1016/s0360-3016(02)02855-9

